# Opinion: The Potential Role of Amyloid Beta Peptides as Biomarkers of Subconcussion and Concussion

**DOI:** 10.3389/fneur.2022.941151

**Published:** 2022-07-11

**Authors:** Angela M. Boutté, Bharani Thangavelu, John Anagli

**Affiliations:** ^1^Aries Biotechnologies, Oakland, CA, United States; ^2^Independent Researcher, Silver Spring, MD, United States; ^3^NeuroTheranostics, Inc., Detroit, MI, United States

**Keywords:** concussion, subconcussion, mild traumatic brain injury, amyloid beta, biomarker, blast

## Introduction

Concussion, often referred to as mild traumatic brain injury (mTBI), is a bump or blow to the head that causes damage to the brain. An invisible wound is often not observed (https://www.cdc.gov/headsup/basics/concussion_whatis.html) (1). Concussed patients may experience loss of consciousness, dizziness, headaches, blurred vision or tired eyes (2, 3), ringing in the ears, bad taste in the mouth, fatigue or lethargy, a change in sleep patterns, behavioral or mood changes, trouble with memory, and slowed reaction time (4, 5). These symptoms may occur acutely or persist for years.

Subconcussion or concussion may occur as the result of high impact sports, like soccer (6), football (7), rugby (8), and gymnastics (9). This injury also occurs due to falls, automobile accidents, and domestic abuse (10, 11), which is vastly understudied. Another source of subconcussion is due to low level overpressure (LLOP) exposure, which occurs when the pressure in air exceeds that of normal atmospheric levels. Exposure is common among specially trained military or law enforcement personnel due to explosives or weapons use within controlled environments. LLOP exposure may also occur within civilian populations who are victims of terrorist bombings (12).

Symptoms reported after concussion or sub-concussive events, e.g., LLOP exposure or a light head hit, are often subjective or under-reported. When symptoms are reported, they often dissipate and are considered a metric of recovery. Subconcussion, in particular, leads to few, low grade, or fleeting symptoms that do not meet the threshold for diagnosis during clinical examination [https://concussionfoundation.org/cte-resources/subconcussive-impacts]. Long-term effects of subconcussion (e.g., per LLOP exposure history and service records) include increased risk of a definitive TBI diagnosis. Subconcussive event history and diagnosed concussion are associated with higher likelihood of neurodegenerative diseases, such as chronic traumatic encephalopathy (CTE) (13), Alzheimer's Disease (AD) (14), and Parkinson's Disease (PD) (15, 16).

### Recent Findings Infer Aβ Has Utility as a Biomarker of Subconcussion and Concussion

Injured persons are frequently characterized as recovered and healthy to resume life activities, rendering identification of long-term effects challenging. Objective classification of sub-concussion before clinical manifestation or neurodegenerative disease diagnosis may offer a pre-text for monitoring. To address this gap, several blood-based protein biomarkers have been evaluated in the context of diagnosed concussion then successfully applied to subconcussive states (17).

Among the milieu of peripheral biomarkers, the most prolifically studied are glial fibrillary acidic protein (GFAP)—an abundant intermediate filament protein enriched in astrocytes; and ubiquitin carboxy hydrolase (UCH)-L1–a cell body enzyme found in neurons. GFAP and UCH-L1 were the first blood-based biomarkers to be approved by the FDA for monitoring TBI (18). Increased levels of GFAP is well-documented among concussed patients (19). However, it offers poor specificity for detection of subconcussive injury, for which damage to the brain is not evident *via* high resolution medical imaging (20).

Amyloid beta peptides (Abeta)−40 and −42 peptides are toxic cleavage products of amyloid precursor protein (APP) enriched within neuronal cell populations. These toxic monomers are crucial to pathogenesis of chronic neurodegenerative diseases (21) and a pathological hallmark of the Alzheimer's disease (AD)-afflicted brain (22). Similar to neurodegenerative disease, severe TBI leads to APP loss with generation of Abeta. APP processing occurs through two major pathways. Non-amyloidogenic processing occurs *via* alpha (α)-secretase and the ADAM family of proteases (23). Whereas, the amyloidogenic pathway relies upon protease activity lent by β-secretase (BACE1) and the gamma (γ)-secretase complex (24–26). Recent studies have indicated cathepsin B beta-secretase activity also leads to APP loss with concomitant Abeta peptide generation in the brain, followed by efflux into cerebrospinal fluid (CSF) and blood (27, 28).

Abeta production and efflux has been reported to occur after subconcussion and concussion. A ProQuest search of publicly available data using the key words, “amyloid beta,” “blood,” “biomarker” and “concussion,” resulted in 1,171 publications, 236 of which were peer-reviewed, from 2017–2022. Peptide quantitation in peripheral blood has become an increasingly popular research tool as an outcome measure of subconcussion, as well as diagnosed concussion.

Hours or days after concussion, Abeta peptides accumulate in increase exorbitantly in peripheral blood. For example, quantitation of Abeta-42 within neuronal exosomes isolated from plasma revealed that was this biomarker elevated among patients who had a history of TBI with impaired cognition (29). This effect was also observed among student athletes who were diagnosed with an mTBI, or concussion. Further, exosomal Abeta-42 nearly doubled compared to controls measured within acute (7 days) and chronic (3 months) time frames (30). Military personnel who suffered an mTBI within <2 years prior to blood collection, showed that Abeta was increased by nearly two-fold within purified exosomes, without association with chronic symptoms (30). In patients or among persons exposed to a subconcussive event, e.g., LLOP, Abeta levels persist in blood for months after the injury incident (31).

High serum Abeta−40 and −42 concentrates were recently associated with subconcussion as demonstrated within several successive reports conducted with military personnel exposed to LLOP. Abeta−40 and −42 were both increased within hours, after daily LLOP exposure from high caliber weapons usage (32). Abeta peptide levels were consistently elevated after LLOP exposure among personnel who reported symptomology either acutely (33) or chronically (34). Serum Abeta levels were nearly 50 times higher among study participants compared to controls who did not endure LLOP exposure in their history. Notably, reports of long-term dizziness, tinnitus (ear-ringing) and memory problems were common among the subconcussion cohort who had the highest Abeta levels in blood. These observations were unambiguous, but did not meet the threshold for clinical diagnosis.

Abeta is associated with concussion, subconcussion and presence of symptoms. However, it is not yet known if discrete quantitative levels, or relative changes over time, are directly proportional to symptom severity or act as prognostic indicators of long-term health outcomes. Large scale, longitudinal studies would address this knowledge gap and offer greater clinical utility.

## Discussion

Abeta measurements as a consequence of subconcussion and diagnosed concussion are evident. However, this feature is not without a few caveats. First, the sample size in most observational studies is fairly small, and the patient and human subject volunteers populations studied are largely composed of Caucasian males. Relevance of Abeta as a biomarker for subconcussion and concussion would be vastly strengthened by analysis of blood from women and a broader mixture of persons from various ethnic or cultural backgrounds. This approach would potentially provide a means to justify clinical imaging and medical monitoring for those who may suffer an accidental fall, or those who may be victims of short and long-term physical abuse. Additionally, monitoring amyloid beta among military personnel or law enforcement may offer a means to modify training guidelines that allow improved health among veterans later in life.

Second, recent studies utilized digital enzyme-linked immunosorbent assays (ELISA) provided by the SiMoA platform as a singular digital technology, which offers broad dynamic range and low limits of detection and quantitation for blood biomarkers. Reported differences in blood Abeta peptide concentrations have been generally reproducible relative to controls. Yet, Abeta concentrations may differ in plasma, compared to serum as well as exosomes (35). Cross-validation has yet to be conducted using these three biological sources of Abeta within the same cohorts. Additionally, validation in comparison other platforms, such as mass spectrometry (36), prior to adaptation for clinical use is needed.

Lastly, L1-CAM is commonly used to enrich exosomes of neuronal origin, since it is enriched within the cerebral cortex and cerebellum. However, this receptor is also detectable within the kidney and epidermis (https://www.proteinatlas.org/ENSG00000198910-L1CAM/tissue). Subconcussion and concussion affect multiple organs. Thus, greater specificity for neuronal exosome isolation and exclusion of damage to other organs, such as the kidney or skin, may offer improved utility of exosomal Abeta as it relates to the subconcussed or concussed brain.

## Conclusions

An uptick in Abeta peptide concentrations, from plasma, serum or exosomes after subconcussion or clinically diagnosed concussion has generally been consistent across studies. Data collectively infer that Abeta−40 and −42 may be more sensitive than that of self-reported symptoms, which are underreported or may dissipate over time.

## Author Contributions

All authors listed have made a substantial, direct, and intellectual contribution to the work and approved it for publication.

## Conflict of Interest

AB is the founder and owner of Aries Biotechnologies, Oakland, CA and an employee of renegade.bio, Berkeley, CA. BT is an employee of Novavax Inc., Gaithersburg, MD. JA is the founder and owner of Neurotheranostics, Inc., Detroit, MI. Neither renegade.bio or Novavax, Inc. had a role in this work.

## Publisher's Note

All claims expressed in this article are solely those of the authors and do not necessarily represent those of their affiliated organizations, or those of the publisher, the editors and the reviewers. Any product that may be evaluated in this article, or claim that may be made by its manufacturer, is not guaranteed or endorsed by the publisher.
